# Predictors Associated With Post-Traumatic Hydrocephalus in Patients With Head Injury Undergoing Unilateral Decompressive Craniectomy

**DOI:** 10.3389/fneur.2018.00337

**Published:** 2018-05-14

**Authors:** Qianxin Hu, Guangfu Di, Xuefei Shao, Wei Zhou, Xiaochun Jiang

**Affiliations:** Department of Neurosurgery, Yijishan Hospital, Wannan Medical College, Wuhu, China

**Keywords:** post-traumatic hydrocephalus, decompressive craniectomy, traumatic brain injury, risk factor, unilateral

## Abstract

**Objective:**

Post-traumatic hydrocephalus (PTH) makes recovery from head trauma after decompression more complicated and is associated with high risks of clinical deterioration and poor outcomes. The aim of this study was to verify the predictors associated with the development of PTH in patients with head injury undergoing unilateral decompressive craniectomy (DC).

**Methods:**

Among traumatic brain injury (TBI) patients who underwent unilateral DC between January 2013 and December 2016, the clinical medical records, radiological information, and changes of patients’ conditions in the 3-month after injury were reviewed retrospectively.

**Results:**

183 TBI patients after unilateral DC were analyzed, and 50 (27.32%) of them suffered PTH based on head CT scans. Univariate and multivariable analyses revealed that older age (*p* = 0.002), the Glasgow Coma Scale (GCS) score at admission (*p* < 0.001), intraventricular hemorrhage (IVH; *p* = 0.008), post-traumatic cerebral infarction (PCI; *p* = 0.007), and postoperative meningitis (*p* = 0.016) were independent predictors for the hydrocephalus after DC. Receiver operating characteristic curves were created and the area under the curve (AUC) were calculated to further assess the accuracy of the variables for predicting PTH. The AUC was 0.836 for the combined all five independent factors (95% confidence interval: 0.775–0.887).

**Conclusion:**

TBI patients who undergo unilateral DC with advanced age, lower GCS score at admission, coexisting IVH, PCI, and/or postoperative meningitis should be closely monitored at follow-up assessments for earlier prediction of PTH.

## Introduction

Previous studies have proved decompressive craniectomy (DC) was an independent risk factor of post-traumatic hydrocephalus (PTH) after a traumatic brain injury (TBI) (1–3). PTH occurs in 11.9–36.0% of patients who undergo DC, which is a concern because PTH can lead to unfavorable outcomes (4–6). PTH is typically characterized by progressive accumulation of cerebrospinal fluid (CSF) and ventriculomegaly secondary to disorders involving CSF circulation and malabsorption. PTH can disrupt brain function or metabolism, delay the clinical improvement, and aggravate TBI outcomes if not be detected and effectively treated in time (7). Therefore, the early diagnosis and treatment of PTH can prevent further neurological complications in patients who are recovering from TBI. However, the findings of studies that have investigated the risk factors associated with PTH after DC are heterogenous due to different evaluation methods and enrollment criteria used.

Thus, the present study performed a retrospective review of patients undergoing unilateral DC to determine the incidence of PTH during hospitalization in the 3 months after TBI. In addition, the prognostic values of the clinical risk factors related to PTH were evaluated, and a predictive receiver operating characteristic (ROC) model was developed by combining the independent predictors associated with PTH.

## Patients and Methods

### Patient Characteristics

This retrospective study was approved by the Institutional Ethical Board of the Yijisha Hospital of Wannan Medical College. The institutional database was searched to identify consecutive TBI patients who underwent unilateral DC and were treated between January 2013 and December 2016. To avoid confounding variables, the following exclusion criteria were applied: patients who had (1) a past neurological history, (2) dilated ventricles or hydrocephalus present on the initial computed tomography (CT) scans or the first CTs were scanned longer than 6 h after injury, (3) undergone a bilateral or bifrontal craniectomy, (4) lost to follow-up, and (5) multiple injuries (abbreviated injury score ≥ 3). Electronic medical records, admission, and follow-up head CT scans were reviewed by two authors. Patients with different diagnosis were also excluded.

### Measured Variables

The following factors were assessed prior to DC and included in the analyses: Glasgow Coma Scale (GCS) scores at admission, hypoxemia, pupil reactivity, status of basal cistern, extent of midline shift, and the presence of traumatic subarachnoid hemorrhage (tSAH), intraventricular hemorrhage (IVH), subdural hemorrhage (SDH), epidural hemorrhage (EDH), and contusion-associated hemorrhage. Hypoxemia was defined as (1) when hemoglobin oxygen saturation was less than 90% or (2) when the partial pressure of oxygen in blood was less than 60 mm Hg at admission. Pupil reactivity was divided into no reactive, one reactive (include “sluggish”), and both reactive. Midline shift was measured in millimeters as a continuous variable. Basal cistern compression was defined as basal cistern narrowing or disappearance, it was dichotomized as “yes” (compressed) and “no” (absent). Potential risk factors after DC were also analyzed and included craniectomy size, decompression time after injury, reoperation, post-traumatic cerebral infarction (PCI), postoperative meningitis, craniectomy margin from midline, various types of hygromas, and Glasgow Outcome Scale Extended (GOSE) at 3 months after TBI. Diagnosis of IVH can be confirmed by the presence of blood inside the ventricles with Graeb scale >1 on CT (8). PCI was defined as low-attenuation lesions in well-defined arterial vascular distribution on any brain CT scan within 2 weeks after the accident, and a diagnosis of cerebral infarction was revised if follow-up studies indicated the findings were actually related to evolving contusions, artifacts, or were inconsistently visualized (9). The definition of postoperative meningitis must meet at least one of the followings: either (1) patient has organisms cultured from CSF or (2) patient has the following signs or symptoms with no other recognized cause: fever (>38°C), meningeal signs and at least one of the followings: (a) increased white cell count, elevated protein, and/or decreased glucose level in CSF. (b) organisms seen on Gram’s stain of CSF (10). Craniectomy size was calculated by skull X-ray for locating the head CT that was taken postoperatively, as described previously (6, 11): craniectomy size = (largest transverse diameter × vertical diameter perpendicular to largest transverse diameter) × π/4. The craniectomy margin from the midline was defined as the mean of the maximum and minimum distances from the midline to the medial border of the bone flap (11). The GOSE was employed to evaluate patients at the end of 3-month follow-up.

### Patients’ Treatment

After admission, patients were initially scanned by CT following serial neurological examinations. When hypoxemia occurs, intubation and mechanical ventilation may be needed. Intracranial traumatic lesions associated with a midline shift >5 mm, volume hematomas ≥30 ml, basal cistern compression, abnormal pupillary reaction, or neurological deteriorations were treated with osmotic therapy or DC. Operative records were reviewed to determine surgical indications and details. There were two clinical scenarios in which DC for TBI was performed. The first scenario involved patients who had DC as part of an operation to treat a hematoma or edema or resective surgery for diffuse injury immediately after admission (“primary DC”). The second was that patients had DC for refractory malignant brain swelling despite maximal medical management (“secondary DC”). Any clinical deterioration indicated the need for another CT scan, and a ventriculoperitoneal shunt might be considered in patients with PTH. All patients were evaluated and treated as prescribed by currently accepted international guidelines (12).

### Definition of PTH

All patients underwent follow-up assessment that included a neurological examination and brain CT scan at 3 months after the injury. The presence of PTH on the serial CT images was featured using enlargement of lateral and third ventricle or as a frontal horn index ≥33% with “infiltration syndrome” around ventricle on CT which was not found before (13, 14);. The clinical features of PTH included high flap-tension and lowering/no improvement of consciousness. Once the patients were suspected of PTH, temporary CSF drainage test was conducted, if the GCS of the patients improved ≥2, we defined them as PTH.

### Statistical Analysis

IBM SPSS Statistics 23 was employed for analyses. *T* tests or Mann–Whitney *U* tests were performed to analyze continuous variables, and chi-square tests were conducted to analyze categorical ones. Significant factors in the univariate analysis were enrolled in a logistic regression to screen independent risk factors and then used in a predictive ROC model. The area under the curve (AUC) values in the ROC analysis were used to assess the discriminatory power of the model, which was classified as follows in terms of predictive ability: good to excellent (AUC > 0.8), moderate (AUC: 0.7–0.8), and low (AUC: 0.6–0.7). Comparison of ROC curves was analyzed by the MedCalc statistical software. *p* Values <0.05 were considered to indicate statistical significance.

## Results

A total of 183 TBI patients who underwent unilateral DC between January 2013 and December 2016 were included in the final analyses. The baseline demographic, clinical, and radiological data of the patients are shown in Table 1. During the 3-month follow-up period, 50 patients (27.32%) developed PTH. At the diagnosis of 50 PTH patients, urinary incontinence was found in 16; increased spasticity in 21; and epileptic seizures in 11. Once the patients were diagnosed as PTH, VP shunt was considered. However, only parts of patients (22/50, 44%) underwent the VP shunts, others did not because of fever, pneumonia, intracranial or urinary tract infection, or high treatment costs. Follow-up in shunted patients showed symptomatic improvements in 17/22 (improvements of urinary incontinence in 6 out of 7; reductions of limb spasticity in 8 out of 9; and reliefs of epileptic seizures in 3 out of 6). In our trauma center, cranioplasty is traditionally conducted at more than 3 months after TBI (15, 16), only 4 of 183 patients underwent cranioplasty within 3-month follow-up. The patients who developed PTH were significantly older (*p* = 0.01) and had significantly different mean GCS scores at admission and hypoxemia and pupil reactivity statuses prior to DC, compared with those who did not develop PTH. Of the coexisting hemorrhages identified on the initial head CT scan, IVH occurred in 88.2% of cases and was significantly associated with PTH (*p* = 0.001). In addition, the PTH and non-PTH groups significantly differed in terms of basal cistern status (*p* < 0.001), midline shift (*p* = 0.025), and craniectomy size (PTH group: 79.38 ± 16.83 cm^2^, non-PTH group: 72.77 ± 16.41 cm^2^; *p* = 0.017).

**Table 1:** Demographic, clinical, and radiological data for 183 patients who underwent DC procedures after traumatic brain injury.

		PTH		*p*
No. of patients	Total (183)	Yes (50)	No (133)	
Mean age in years (SD)	53.04 ± 14.36	57.46 ± 13.76	51.38 ± 14.27	0.01
Sex				0.214
Female	50	17	33	
Male	133	33	100	
GCS scores on admission (SD)	7 ± 2	6 ± 2	7 ± 2	<0.001
Hypoxemia				0.001
Yes	28	15	13	
No	155	35	120	
Pupil reactivity				<0.001
None	47	23	24	
One reactive	31	12	19	
Both reactive	105	15	90	
Type of DC				0.453
Primary DC	74	18	56	
Secondary DC	109	32	77	
SAH				0.125
Yes	161	47	114	
No	22	3	19	
IVH				<0.001
Yes	22	13	9	
No	161	37	124	
SDH				0.667
Yes	143	38	105	
No	40	12	28	
EDH				0.632
Yes	41	10	31	
No	142	40	102	
Contusion-associated hemorrhage				0.172
Yes	158	46	112	
No	25	4	21	
Status of basal cistern				<0.001
Absent	70	30	40	
Compressed	113	20	93	
Midline shift (mm)	9.2 ± 5.0	10.5 ± 5.0	8.7 ± 4.9	0.025
Craniectomy size (cm^2^)	74.57 ± 16.74	79.38 ± 16.83	72.77 ± 16.41	0.017
Decompression time (hours)				0.074
<6 h	100	34	66	
6 h to <24 h	52	11	41	
24 h≥	31	5	26	
Reoperation				0.544
Yes	35	11	24	
No	148	39	109	
PCI				0.001
Yes	28	15	13	
No	155	35	120	
Craniectomy margin from midline (cm)				0.076
≦2.5	83	28	55	
>2.5	100	22	78	
Subdural hygroma				0.055
Yes	115	37	78	
No	68	13	55	
Interhemispheric hygroma				0.013
Yes	25	12	13	
No	158	38	120	
Bilateral hygroma				0.678
Yes	9	3	6	
No	174	47	127	
Ipsilateral hygroma				0.277
Yes	74	17	57	
No	109	33	76	
Contralateral hygroma				0.026
Yes	13	7	6	
No	170	43	127	
Postoperative meningitis				0.008
Yes	7	5	2	
No	176	45	131	
GOSE (SD)	5 ± 2	3 ± 2	6 ± 2	<0.001
Shunted (22)	–	4 ± 2	–	
Non-shunted (28)	–	2 ± 0	–	

*GCS, Glasgow Coma Scale; tSAH, traumatic subarachnoid hemorrhage; IVH, intraventricular hemorrhage; SDH, subdural hemorrhage; EDH, epidural hemorrhage; PCI, post-traumatic cerebral infarction; GOSE, Glasgow Outcome Scale Extended; PTH, post-traumatic hydrocephalus; DC, decompressive craniectomy*.

Of the variables measured after DC, the incidences of PCI and postoperative meningitis were significantly higher in the PTH group compared with the non-PTH group (*p* = 0.001 and *p* = 0.017, respectively). However, interhemispheric hygroma was the only factor associated with PTH among all types of hygromas (*p* = 0.017). The GOSE scores of the PTH patients were significantly lower at the 3-month post-injury assessment compared with the non-PTH patients (*p* < 0.001). The multivariable analysis revealed that older age (*p* = 0.002), GCS score at admission (*p* < 0.001), and the presence of IVH (*p* = 0.008), PCI (*p* = 0.007), and postoperative meningitis (*p* = 0.016) were independently associated with PTH after DC (Table 2).

**Table 2:** Multiple logistic regression model for factors associated with PTH.

Factors	OR	95% CI	*p* Value
Age	1.052	1.109–1.087	0.002
GCS scores on admission	0.651	0.527–0.803	<0.001
IVH	4.091	1.435–11.658	0.008
PCI	4.059	1.469–11.214	0.007
Postoperative meningitis	11.876	1.578–89.388	0.016

*OR, odds ratio; CI, confidence interval; GCS, Glasgow Coma Scale; IVH, intraventricular hemorrhage; PCI, post-traumatic cerebral infarction; PTH, post-traumatic hydrocephalus*.

**Figure 1:**
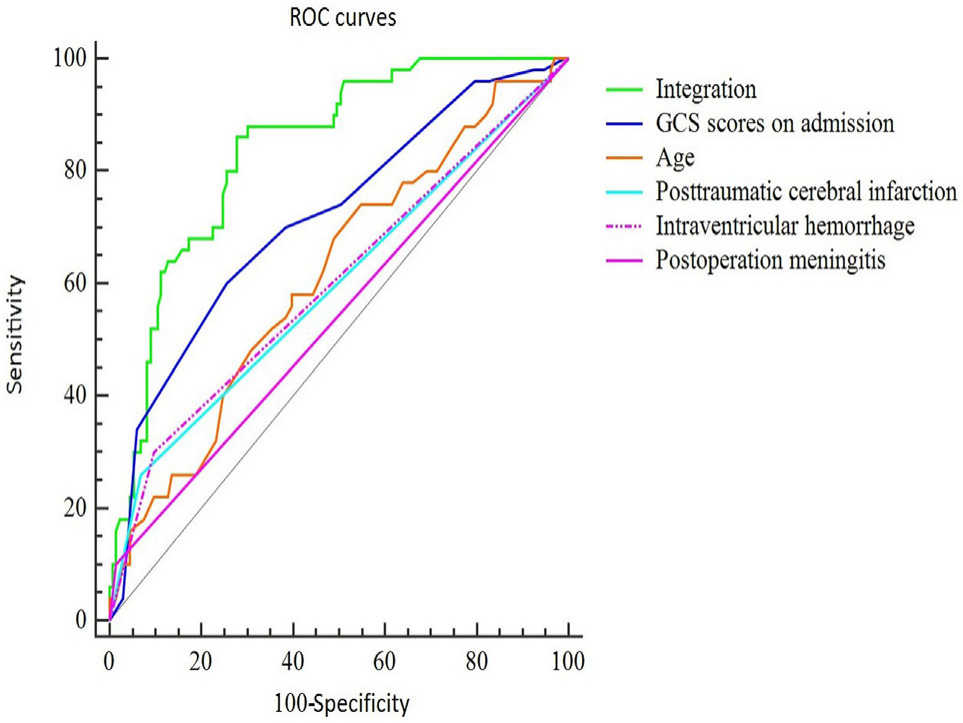
ROC curves of the final predictive model using five main risk factors. The sensitivity and specificity of the final combined curve were 0.86 and 0.72, respectively. Abbreviations: GCS, Glasgow Coma Scale; IVH, intraventricular hemorrhage; PCI, post-traumatic cerebral infarction.

To further assess the accuracy of the factors that may predict PTH, ROC curves were created, and the AUC values were calculated (Figure 1). The AUC was 0.615 [95% confidence interval (CI): 0.540–0.685] for age (with a cut-off value of > 51 years), 0.715 (95% CI: 0.644–0.779) for GCS scores at admission (with a cut-off value of < 6), 0.596 (95% CI: 0.521–0.668) for IVH, 0.601 (95% CI: 0.526–0.673) for PCI, and 0.542 (95% CI: 0.467–0.616) for postoperative meningitis (Table 3). Finally, the AUC for these five factors combined was 0.836 (95% CI: 0.775–0.887), with a sensitivity of 86% and a specificity of 72%. These findings indicate that the combined five factors exhibit excellent predictive power for PTH.

**Table 3:** The AUCs under the ROC curves.

	AUC	SE	95% CI
Combined	0.836	0.0310	0.775–0.887
Age	0.615	0.0468	0.540–0.685
GCS scores on admission	0.715	0.0432	0.644–0.779
IVH	0.596	0.0332	0.521–0.668
PCI	0.601	0.0352	0.526–0.673
Postoperative meningitis	0.542	0.0221	0.467–0.616

*AUC, area under the curve; ROC, receiver operating characteristic; GCS, Glasgow Coma Scale; IVH, intraventricular hemorrhage; PCI, post-traumatic cerebral infarction; CI, confidence interval*.

## Discussion

Post-traumatic hydrocephalus is a common and devastating complication that may occur following TBI. Although various studies have reported the incidence of PTH (1, 17), few have specifically analyzed TBI patients who underwent unilateral DC or focused on the risk factors for PTH in this subgroup. Thus, the present study diagnosed PTH according to changes of imaging or clinical features and identified a PTH incidence of 27.32%. Moderate and severe TBI patients without DC between January 2013 and December 2016 in our center were also reviewed and only 9 out of 112 (8.0%) developed a PTH at 3-month post-injury. These results showed this sub-cohort might actually be more susceptible to PTH. In our trauma center, more than 300 patients with moderate-to-severe TBI are admitted each year. PTH may be easily overlooked during rehabilitation and its consequences are extremely destructive. Thus, early prediction of hydrocephalus will be valuable for TBI evaluation after DC and for improved outcomes. In the present study, univariate and multivariable analyses were conducted to determine the risk factors associated with PTH after DC; the independent predictive factors included older age, lower GCS score at admission, and the presence of IVH, PCI, and postoperative meningitis after DC. The ROC curve for these five factors integrated provided good discrimination of PTH risk, with an AUC that was indicative of excellent predictive power.

Advanced age is an influential factor in the development of PTH in TBI patients (1, 17). Older patients have a wider subarachnoid space that can hold larger volumes of subarachnoid blood, which increases their risk of developing disturbances in CSF circulation (18). It is also possible that the extent of meningeal fibrosis, which impairs CSF circulation and decreases CSF absorption, is greater in elderly patients (19). In the present study, the ROC for the age determined the cut-off value of the age was 51, which means when the age >51 years, the probability of PTH significantly increased. A lower GCS score at admission was also a risk factor of hydrocephalus in our study, which is in correspondence with prior findings (1, 5, 17). Patients with lower GCS scores or severe consciousness disorders generally suffer from grave disturbances in CSF formation or absorption and increasing the risk of development of hydrocephalus.

Other potential risk factors for PTH include various types of coexisting hemorrhages evident on a CT scan (2, 20). However, in the present study, only IVH was significantly associated with PTH, while tSAH, SDH, and EDH were not. Severe IVH after TBI promotes the formation of hydrocephalus and abnormal CSF flow through an obstructive mechanism. Small blood clots or other substances can obstruct CSF circulation at the arachnoid granulation site or block the fourth ventricle or sacra fistula directly, which would cause decreased CSF absorption, increased CSF accumulation, and ultimate formation of PTH (21).

The craniectomy size was bigger in the PTH group, it has been suggested that craniectomy may play a role in the flattening of the normal dicrotic intracranial waveform due to the transmission of the pressure pulse out through the open cranium, which leads to decreased CSF outflow to the arachnoid granulations (11, 13). Thus, a bigger craniectomy size may be a risk factor for the development of hydrocephalus, *via* an impairment of hydrodynamic CSF circulation. In the PTH patients, another important reason of using a larger craniectomy may be more severe injury or lower admission GCS scores. The real mechanism of its effect on PTH remains unclear. According to our results, the craniectomy size did not affect the development of PTH independently, but we should not neglect its role in future study.

In the present study, the incidence of PCI was significantly higher in the PTH group than in the non-PTH group and was an independent risk factor for hydrocephalus. PCI is a severe and common complication in patients with head trauma, and blunt cerebral vascular injuries, hypotension, high ICP, low GCS scores, brain herniation, and DC may be risk factors for PCI in patients with moderate or severe TBI (9, 22). Cerebral infarctions after TBI may lead to reduced regional cerebral perfusion as well as microangiopathy, hypoxia, necrosis, and even neurological deficits. In addition, in communicating hydrocephalus, disorders involving the CSF and cerebrovascular circulation are associated with one another (23, 24), but the causal link between these factors remains unknown. Peña and colleagues (23, 25) proposed that the ventricular dilatation associated with communicating hydrocephalus results from a reversal of interstitial fluid flow into the parenchyma and reduced tissue elasticity. This may explain the relationship between PCI and PTH.

Postoperative meningitis or infections may be among the most common causes of hydrocephalus (26), which is supported by the present results. Chen et al. (1) found no evidence of a correlation between intracranial infections and PTH, but this may have been due to the use of continuous lumbar CSF drainage in their patients. A prior study (2), which demonstrated that the continuous lumbar subarachnoid drainage significantly reduces the hydrocephalus after TBI, confirmed this deduction.

Finally, an ROC model was created in the present study to further assess the accuracy of these factors for predicting PTH. Based on the AUC values, only the combined all five factors provided a good discrimination of PTH risk, and the AUC was indicative of excellent predictive power. Independently, GCS score at admission had a moderate predictive accuracy, whereas the other factors (age and the presence of IVH, PCI, and postoperative meningitis) had low predictive values.

Traumatic brain injury is still a main reason of morbidity and disability among trauma groups, which is also responsible for a notable ratio of all traumatic deaths. The disability and loss of human potential and long-term impairment resulted from TBI has led to enormous influence to the society and family as well as costs society a significant amount of money each year. In our trauma center, once there is a surgical indication, immediate DC is performed to save the patient’s life in despite of lack of money. If the injury was caused by traffic accident, the person who caused the accident will pay the victim. TBI caused by other reason may be paid by their families, or their social and commercial insurance agents. The high medical costs and insufficient insurance have brought the financial burden to the poor families. Unfortunately, some of them may postpone or opt out from the beneficial treatments. Although early prediction and diagnosis of PTH may help to decrease the hospital days and expenses, our government and community may still need to take effective measures, such as increasing the use of safety belts, and increasing personal protection for dangerous work to prevent and reduce the incidence of TBI.

There were several limitations to this study. First, due to only parts of PTH patients underwent the VP shunts, relatively few cases of VP-shunt-conducted hydrocephalus limited the power to identify risk factors for the development of VP-shunt-conducted PTH. Second, this was a retrospective study based on neurological examinations and radiological findings that had a limitation of evaluating whether patients might benefit from shunt operation for CSF diversion. Therefore, future research should focus on recruiting more cases, and should implement long-term follow-up and a more thorough review of patients in a prospective manner.

## Conclusion

The present study demonstrated that the risk of PTH can be independently predicted by older age, lower GCS score at admission, PCI, IVH, and intracranial infection after DC. However, a combination of these five factors provided much stronger predictive power. In the future, comprehensive analyses should be conducted in high-risk TBI patients undergoing unilateral DC to better understand the relationships among these factors.

## Ethics Statement

This retrospective study was approved by the Institutional Ethical Board of the Yijisha Hospital of Wannan Medical College.

## Author Contributions

QH and XJ designed the experiments; GD and XS performed data analysis; WZ provided scientific expertise; QH wrote the manuscript. Because QH and GD contributed equally to this work, they are considered as co-first authors.

## Conflict of Interest Statement

The authors declare that the research was conducted in the absence of any commercial or financial relationships that could be construed as a potential conflict of interest.
